# Synthesis and anticonvulsant activity of new *N*-phenyl-2-(4-phenylpiperazin-1-yl)acetamide derivatives

**DOI:** 10.1007/s00044-015-1360-6

**Published:** 2015-03-10

**Authors:** Krzysztof Kamiński, Beata Wiklik, Jolanta Obniska

**Affiliations:** Department of Medicinal Chemistry, Faculty of Pharmacy, Jagiellonian University Medical College, 9 Medyczna Street, 30-688 Kraków, Poland

**Keywords:** Phenylacetamides, Anticonvulsant activity, SAR studies, In vivo studies, In vitro studies, Epilepsy

## Abstract

Twenty-two new *N*-phenyl-2-(4-phenylpiperazin-1-yl)acetamide derivatives have been synthesized and evaluated for their anticonvulsant activity in animal models of epilepsy. These molecules have been designed as analogs of previously obtained anticonvulsant active pyrrolidine-2,5-diones in which heterocyclic imide ring has been changed into chain amide bound. The final compounds were synthesized in the alkylation reaction of the corresponding amines with the previously obtained alkylating reagents 2-chloro-1-(3-chlorophenyl)ethanone (**1**) or 2-chloro-1-[3-(trifluoromethyl)phenyl]ethanone (**2**). Initial anticonvulsant screening was performed using standard maximal electroshock (MES) and subcutaneous pentylenetetrazole screens in mice and/or rats. Several compounds were tested additionally in the psychomotor seizures (6-Hz model). The acute neurological toxicity was determined applying the rotarod test. The results of pharmacological studies showed activity exclusively in the MES seizures especially for 3-(trifluoromethyl)anilide derivatives, whereas majority of 3-chloroanilide analogs were inactive. It should be emphasize that several molecules showed also activity in the 6-Hz screen which is an animal model of human partial and therapy-resistant epilepsy. In the in vitro studies, the most potent derivative **20** was observed as moderate binder to the neuronal voltage-sensitive sodium channels (site 2). The SAR studies for anticonvulsant activity confirmed the crucial role of pyrrolidine-2,5-dione core fragment for anticonvulsant activity.

## Introduction

**Fig. 1:**
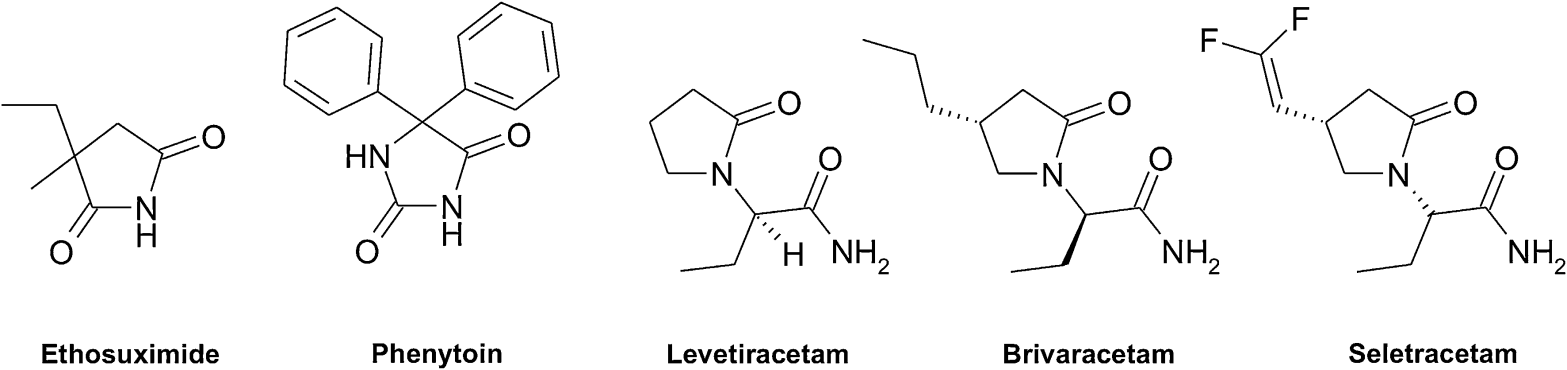
Structures of known AEDs containing nitrogen heterocyclic ring

Epilepsy is one of the most prevalent neurological disorders, affecting approximately 0.5–1 % people worldwide. It has a chronic and progressive nature, characterized by recurring seizures of various manifestations. The basic method of the treatment of epilepsy is pharmacotherapy. It is a symptomatic treatment which allows abolishing or reducing the number of seizure episodes, however, does not inhibit the pathophysiological processes of epileptogenesis and does not eliminate organic changes in the central nervous system (CNS), which are the underlying causes of the disease. In about 25 % of patients, the desired results cannot be achieved with the use of pharmacotherapy, this form of disease is known as refractory epilepsy, the treatment of which is based on surgical vagus nerve stimulation (WHO, 2010; Bell and Sander, 2002; Chang and Lowenstein, 2003). The variety of molecular targets, not always precisely definite, makes it difficult to establish an unequivocal mechanism of action of anti-epileptic drugs (AEDs). This fact is also reflected in the limited possibilities of designing new drugs on the base of the structure of a given biological target. Therefore, currently, there are two different methods of search for new anticonvulsants: modifications of clinically effective AEDs or synthesis of entirely new structures (Khan *et al*., 2012). Taking into consideration the above limitations, many authors conducted attempts to identify the structural features crucial for anticonvulsant activity. On the basis of these researches, several pharmacophoric models, enabling a more rational design of new anticonvulsants, have been described. Thus, one of the important core fragments of anticonvulsants is defined by nitrogen heterocyclic system, usually imide or lactam and phenyl or alkyl groups attached to the heterocyclic system (Wong *et al*., 1986; Bruno-Blanch *et al*., 2003; Malawska, 2005). This common template is present in the structures of old, however well-established AEDs, such as ethosuximide and phenytoin as well as among the newest drugs, e.g., levetiracetam, brivaracetam or seletracetam (Rogawski and Porter, 1990; Bialer *et al*., 2007) (Fig. 1).

**Fig. 2:**
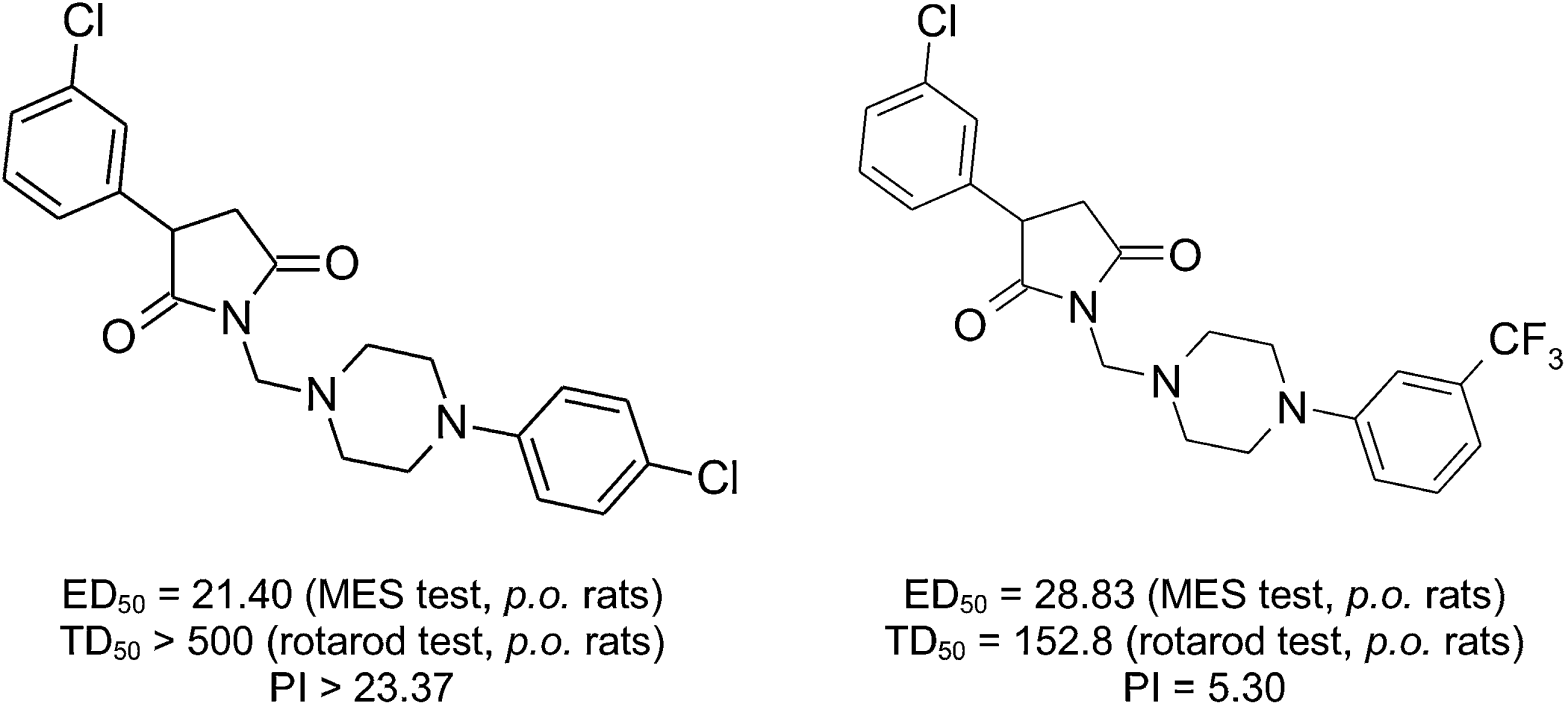
Structures of model pyrrolidine-2,5-diones obtained in the previous research

Bearing in mind the aforementioned, in our laboratory, since many years we have been conducting studies in the group of pyrrolidine-2,5-dione derivatives differently substituted at the imide nitrogen atom as well as at the 3-position of the heterocyclic ring as candidates on new AEDs. Many of these compounds were highly effective in the animal models of epilepsy (Kamiński *et al*., 2014, 2013, 2011a, b; Obniska *et al*., 2013, 2010; Obniska and Zagórska, 2003). Structures and designated median effective doses (ED_50_) in the maximal electroshock (MES) seizure test for the model pyrrolidine-2,5-diones obtained in former research (Obniska *et al*., 2013) are shown in Fig. 2.

**Fig. 3:**
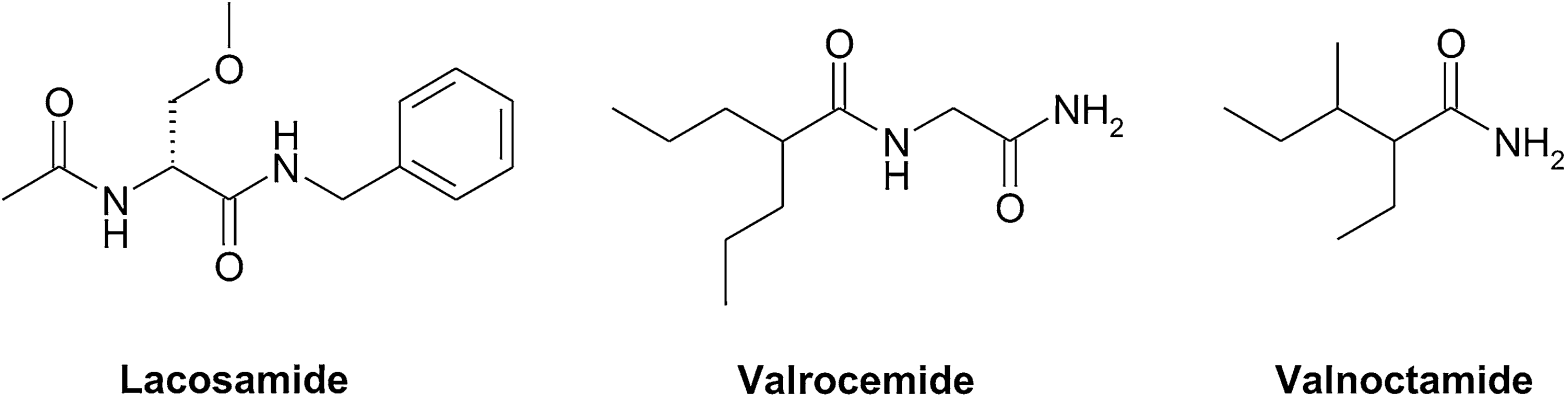
Structures of known AEDs containing chain amide bound

Another important structural fragment of many anticonvulsants as well as compounds being currently in clinical trials is an amide moiety presented in a chain form (Bruno-Blanch *et al*., 2003; Hadjipavlou-Litina, 1998; Luszczki, 2009; Hen *et al*., 2010; Morieux *et al*., 2008; Ghidini *et al*., 2006; Beguin *et al*., 2003; Masereel *et al*., 2002). It should be stressed that this chemical structure possessed several the newest AEDs such as lacosamide (chemically the functionalized amino acid) or valrocemide and valnoctamide which are the unteratogenic derivatives of valproic acid (Fig. 3).

**Fig. 4:**
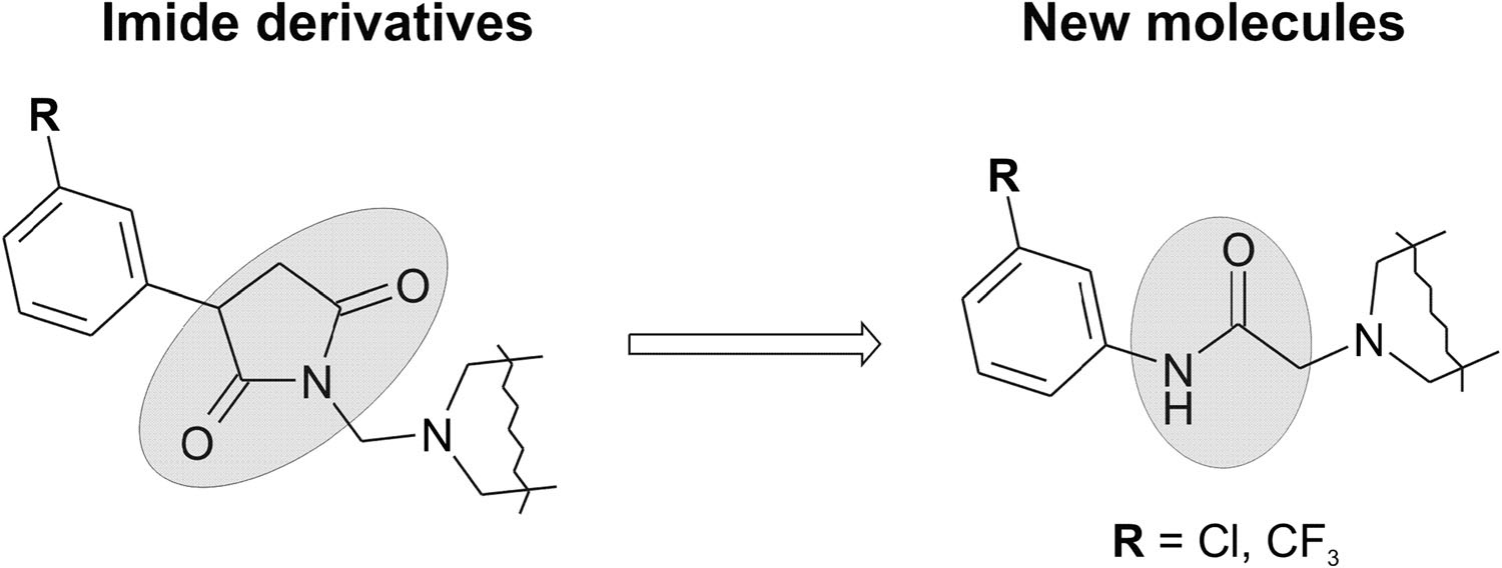
The exchange of the pyrrolidine-2,5-dione core fragment for chain amide bound

Taking into consideration the above findings in the current studies, we have focused on synthesis of new *N*-phenylacetamide derivatives as targets for new AEDs. These compounds were designed as analogs of corresponding pyrrolidine-2,5-dine derivatives, which showed high anticonvulsant activity in the animal models of epilepsy (Obniska *et al*., 2010). The main modification relied on the change of imide ring into chain amide bound (Fig. 4). Therefore, the aim of the current studies was to elucidate how the structure of imide/amide fragment affects anticonvulsant activity.

## Results and discussion

### Chemistry

**Scheme 1:**
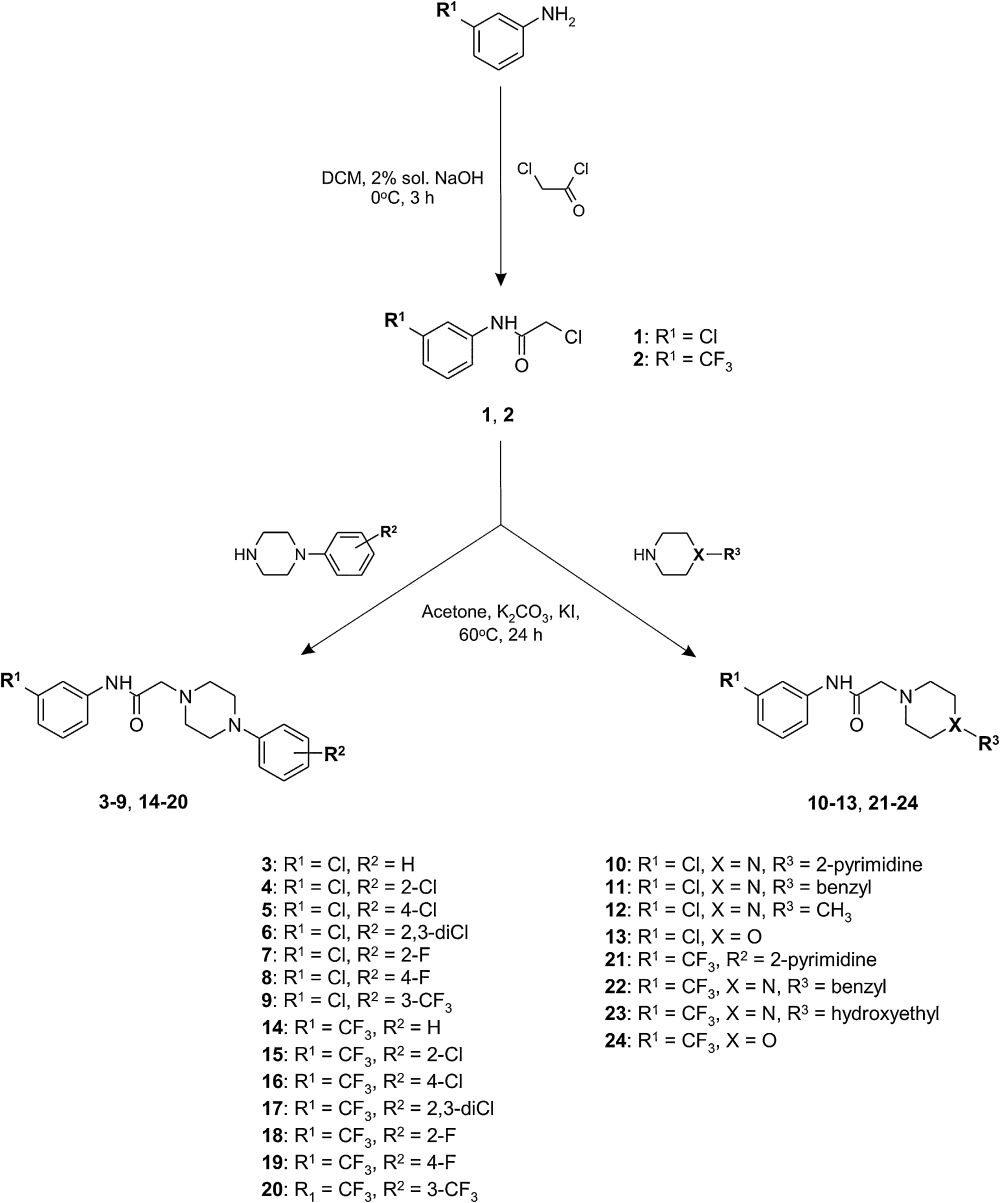
Synthetic pathways of intermediates **1**, **2** and target compounds **3**–**24**

**Fig. 5:**
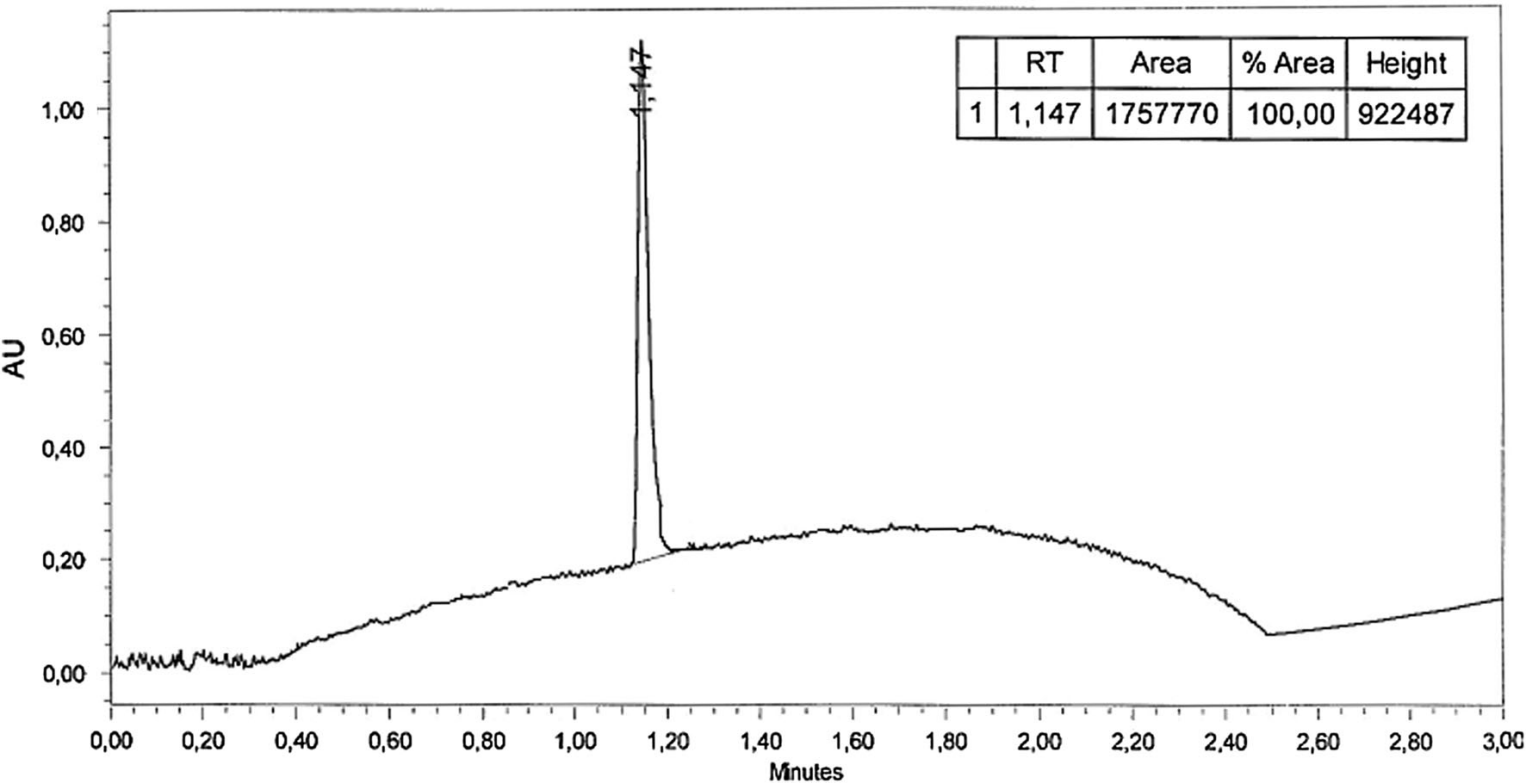
The HPLC chromatogram of compound **22**

The synthesis of compounds **3**–**24** was accomplished as shown in Scheme 1. The starting materials 2-chloro-1-(3-chlorophenyl)ethanone (**1**) and 2-chloro-1-[3-(trifluoromethyl)phenyl]ethanone (**2**) were prepared by acylation of 3-chloroaniline or 3-trifluoromethylaniline with 2-chloroacetyl chloride. The reaction was carried out at 0 °C for 3 h in the mixture consisted of dichloromethane (DCM) and 2 % aqueous sodium hydroxide solution. The final compounds **3**–**24** were synthesized in the alkylation reaction of the corresponding amines with the previously obtained alkylating reagents **1** and **2**. The reaction was carried out at a temperature of 60 °C in a biphasic liquid–solid system consisting of dry acetone, potassium carbonate and catalytic amount of potassium iodide. The progress of the reaction was monitored using HPLC chromatography. Compounds **3**–**24** were obtained in yields ranging from 44 to 78 %. Their purities were assessed by HPLC chromatography (the HPLC chromatogram of **22** is shown in Fig. 5). The final compounds were fully characterized by elemental analyses (C, H, N) and ^1^H NMR, ^13^C NMR, ^19^F NMR, LC/MS spectra. The detailed physicochemical and analytical data are listed in the experimental section.

The ^1^H NMR spectrum of compound **3** displayed several proton chemical shifts consistent with the proposed structure. The eight protons of piperazine moiety were observed as broad multiplet (*δ* 3.06–3.93 ppm). The methylene linker at the acetamide fragment occurred as singlet at *δ* 4.33 ppm. The aromatic protons were observed at the expected chemical shifts as six multiplets within the range of *δ* 6.81–7.86 ppm. The amide (NH) proton appeared as broad singlet at *δ* 10.86 ppm. The proton of protonated piperazine fragment (+NH) was observed as broad singlet at *δ* 11.55 ppm. The ^13^C NMR spectrum of **3** showed four peaks at *δ* 51.3, 51.9, 52.5 and 53.3 ppm which correspond to carbon atoms of piperazine ring. The carbon atom of the methylene linker at the acetamide fragment occurred as peak at *δ* 54.1 ppm. The peaks of carbon atoms of the aromatic rings were detected at the expected chemical shifts within the range *δ* 111.7–146.3 ppm. The peak at 172.1 ppm was ascribed to a carbon in a carbonyl group.

The ^1^H NMR spectrum of **13** revealed two multiplets within the range of *δ* 1.59–1.88 and 2.96–3.56 ppm which were ascribed to eight protons of the morpholine ring. The methylene linker at the acetamide fragment occurred as singlet at *δ* 4.15 ppm. Four aromatic protons gave three multiplets and on triplet within the range of *δ* 7.13–7.86 ppm. The amide (NH) proton appeared as broad singlet at *δ* 10.05 ppm. The proton of protonated morpholine fragment (+NH) was observed as broad singlet at *δ* 11.41 ppm. The ^13^C NMR spectrum of **13** showed five peaks at *δ* 51.8, 52.6, 53.2, 67.8 and 68.6 ppm which correspond to carbon atoms of morpholine ring and the methylene linker at the acetamide fragment. The aromatic carbons in the benzene ring were observed as six peaks within the range *δ* 117.8–138.1 ppm. The peak at 171.5 ppm was ascribed to a carbon in a carbonyl group.

### Anticonvulsant activity

The preclinical development of new chemical agents for the treatment of epilepsy is based on the use of predictable animal seizure models, which correspond to different types of human epilepsies. Despite the diversity of models that could potentially be used to screen for anticonvulsant activity, the MES model and the subcutaneous pentylenetetrazole (*sc*PTZ) model remain the “gold standards” in the early stages of testing. The MES test is the mechanism-independent animal seizure model which enables identification of compounds preventing seizure spread. This test uses an electrical stimulus to produce generalized tonic–clonic seizures and thus is thought to be an experimental model of tonic–clonic epilepsy and of partial convulsions with or without secondary generalization in humans. The *sc*PTZ test employs chemically induced myoclonic seizures and is proposed to identify the agents raising the seizure threshold. This test is related to human generalized absence seizures (Rogawski, 2006).

Considering the aforementioned facts, the profile of anticonvulsant activity of final compounds **3**–**24** was established in the MES and *sc*PTZ tests, after intraperitoneal (*i.p.*) injection in mice at doses of 30, 100 and 300 mg/kg. An observation was carried out at two different time intervals—0.5 and 4 h. Furthermore, in addition to the primary anticonvulsant screening, the acute neurological toxicity (NT) was determined in mice by the rotarod test. The results of the preliminary pharmacological studies are summarized in Table 1.

**Table 1:** Anticonvulsant and neurotoxicity screening after *i.p.* administration in mice (**3**–**24**)

No.	*R* ^1^	*R* ^2^	*X*	*R* ^3^	Intraperitoneal administration in mice^a^						clog *P* ^e^
					MES^b^		*sc*PTZ^c^		NT^d^		
					0.5 h	4 h	0.5 h	4 h	0.5 h	4 h	
**3**	Cl	H	–	–	–	–	–	–	–	–	3.15
**4**	Cl	2–Cl	–	–	–	–	–	–	–	–	3.71
**5**	Cl	4–Cl	–	–	–	–	–	–	–	–	3.71
**6**	Cl	2,3–Cl	–	–	–	–	–	–	–	–	4.26
**7**	Cl	2–F	–	–	–	–	–	–	–	–	3.31
**8**	Cl	4–F	–	–	–	–	–	–	–	–	3.31
**9**	Cl	3–CF_3_	–	–	–	–	–	–	–	–	4.07
**10**	Cl	–	N		–	–	–	–	–	–	1.72
**11**	Cl	–	N		–	–	–	–	300	–	2.80
**12**	Cl	–	N	–CH_3_	100	–	–	–	–	–	1.07
**13**	Cl	–	O	–	100	300	–	–	300	–	0.92
**14**	CF_3_	H	–	–	–	100	–	–	300^Z^	300	3.51
**15**	CF_3_	2–Cl	–	–	–	–	–	–	–	–	4.07
**16**	CF_3_	4–Cl	–	–	–	100	–	–	–	–	4.07
**17**	CF_3_	2,3–Cl	–	–	–	–	–	–	–	–	4.63
**18**	CF_3_	2–F	–	–	–	100	–	–	–	–	3.67
**19**	CF_3_	4–F	–	–	300	100	–	–	300	–	3.67
**20**	CF_3_	3–CF_3_	–	–	–	100	–	–	–	–	4.43
**21**	CF_3_	–	N		–	–	–	–	300	–	2.09
**22**	CF_3_	–	N		300	–	–	–	300^33^	–	3.17
**23**	CF_3_	–	N	–(CH_2_)_2_–OH	300	300	–	–	300	–	0.92
**24**	CF_3_	–	O	–	100	–	–	–	100	–	1.28
Phenytoin^f^					30	30	–	–	100	100	

Response comments: ^Z^ respiratory depression, ^33^ tremors

^a^Doses of 30, 100 or 300 mg/kg were administered intraperitoneally. The data indicate the minimum dose effective or neurotoxic in half or more animals tested. A dash indicates the absence of anticonvulsant activity or neurotoxicity at the maximum dose administered

^b^Maximal electroshock test

^c^Subcutaneous pentylenetetrazole test

^d^Neurotoxicity screening using rotarod test

^e^clog *P* values calculated using a log *P* module of ChemDraw Ultra program, version 7.0.1 (Cambridge Soft Corporation, Cambridge, MA, USA)

^f^Phenytoin, reference antiepileptic drug, tested by use of ADD Program procedures in NIH/NINDS

The compounds tested showed protection exclusively in the MES seizures. Furthermore, in vivo data revealed that anticonvulsant activity was closely connected with the type of substituent at the 3-position of the anilide moiety. Except for *N*-(3-chlorophenyl)-2-(4-methylpiperazin-1-yl)acetamide (**12**)—effective in the MES test at 0.5 h (dose of 100 mg/kg) and *N*-(3-chlorophenyl)-2-morpholino-acetamide (**13**) which showed protection in both time points (100 mg/kg at 0.5 h, and 300 mg/kg at 4 h), all other 3-chloroanilides (**3–11**) were devoid of anticonvulsant activity. As it can be seen, considerably higher anticonvulsant protection was observed for 3-(trifluoromethyl)anilides (**14–24**). Among these derivatives, all compounds, except for those containing 4-(2-chlorophenyl)piperazine (**15**), 4-(2,3-dichlorophenyl)piperazine (**17**) and 4-pyrimidin-2-yl-piperazine (**21**), revealed protection in the MES test at dose of 100 mg/kg and/or 300 mg/kg in different pretreatment times. The highest anticonvulsant activity was observed for **19** which protected animals in both time intervals (300 mg/kg at 0.5 h, and 100 mg/kg at 4 h). Other active compounds with phenylpiperazine fragment as amine function (**14**, **16**, **18**, **20**) revealed protection in the MES seizures only 4 h after *i.p.* administration that means delayed onset, however, long in duration anticonvulsant action. The anti-MES activity at dose of 100 mg/kg showed also morpholine derivative **24** (0.5 h). The differences in times points in which the anticonvulsant protection was observed for compounds containing morpholine (**24**) or phenylpiperazine moieties (**14**, **16**, **18**, **20**) may result from the lipophilic properties of these molecules. As it is presented in Table 1, the more lipophilic molecules (higher clog *P* values)—**14**, **16**, **18**, **20** showed activity mainly at 4 h, whereas less lipophilic compound **24** was effective only at time point of 0.5 h. These observations may be connected with higher affinity of more lipophilic compounds to the peripheral tissues that causes slower distribution to the CNS. The further SAR analysis indicated that exchange of phenylpiperazine moiety into benzylpiperazine (**22**) or hydroxyethylpiperazine (**23**) decreased anticonvulsant activity. The screening data revealed that all active compounds showed weaker anti-MES protection than phenytoin that is still recognized as model AED active in MES test. Furthermore, the anticonvulsant protection was distinctly lower in comparison with respective succinimide analogs described in advance (Obniska *et al*., 2013). This observation confirms the role of pyrrolidine-2,5-dione ring as a pharmacophore crucial for anticonvulsant activity.

The pharmacological results obtained in the current studies proved that introduction of the fluorine atom or trifluoromethyl group was essential for anticonvulsant activity in this series of compounds. Furthermore, it is widely accepted that incorporation of fluorine into biologically active molecules provides analogs with increased metabolic stability due to strength of C–F bond and further reduction of cytochrome P450 oxidative metabolism. Moreover, the unique features of fluorine, such as relatively small size, combined with a very high electronegativity can specifically modulate the steric and electronic properties of the fluorinated derivatives and significantly affect biological activity. In addition, incorporation of fluorine residues into aromatic system leads to increased lipophilicity and better distribution to CNS (Kirk, 2006; Song *et al*., 2005; Ismail, 2002; Smart, 2001). It should be stressed here that beneficial influence of fluorine substitution on anticonvulsant activity was observed in a group of 2-azaspiro[4.4]nonane- and 2-azaspro[4.5]decane-1,3-diones described in advance (Obniska *et al*., 2006).

In the rotarod test (NT) for acute neurological toxicity, most of 3-chloro- as well as 3-(trifluoromethyl)anilides with electron-withdrawing substituents (Cl, F, CF_3_) at the phenylpiperazine fragment did not show neurotoxicity in the maximum dose administered (300 mg/kg). The other derivatives, namely **11**, **13**, **14**, **19**, **21**–**23** showed neurotoxicity at a dose of 300 mg/kg. At the same dose, mice showed respiratory depression after administration of **14** and revealed tremors after administration of **22**. Only one compound **24** revealed neurotoxicity at a dose of 100 mg/kg.

According to the Anticonvulsant Screening Project dispositions, three compounds **13**, **20** and **22** which showed protection in mice were selected and examined for their anticonvulsant activity (MES screen) and neurotoxicity after oral (*p.o.*) administration in rats at fixed dose 30 mg/kg. This screen discloses the time of onset, the approximate time of peak effect (TPE) and the duration of anticonvulsant activity or neurotoxicity. The results are shown in Table 2.

**Table 2:** Anticonvulsant activity of selected compounds administrated orally in rats (MES screen)

No.	Oral administration in rats^a^				
	0.25 h	0.5 h	1 h	2 h	4 h
**13**	0/4	1/4	0/4	0/4	0/4
**20**	0/4	1/4	0/4	2/4	0/4
**22**	1/4	1/4	1/4	1/4	1/4
Phenytoin^b^	1/4	4/4	3/4	3/4	3/4

^a^Dose of 30 mg/kg was administrated orally. The data indicate: number of rats protected/number of rats tested

^b^Phenytoin, reference antiepileptic drug, tested by use of ADD Program procedures in NIH/NINDS

Among derivatives tested, only **20** was active in 50 % of animals at time point 2 h. This compound was also active in 25 % of animals at 0.5 h. The other molecules revealed 25 % protection at 0.5 h (**13**) and in all five time intervals (**25**). All compounds given orally did not cause motor impairment in the rotarod screen (data not indicated in Table 2). The compounds tested showed lower activity than phenytoin which protected from 25 to 100 % of mice in different time intervals.

On the basis of the preliminary data in rats, compound **20** was chosen for quantification of the pharmacological parameters (ED_50_ and TD_50_) after *p.o.* application. The quantitative evaluation of the MES median effective dose (ED_50_) and toxic dose (TD_50_) was performed at previously estimated TPE—2 h. The results of the quantitative tests along with data for the standard AEDs—valproic acid, ethosuximide and phenytoin—are listed in Table 3.

**Table 3:** Quantification data in rats after *p.o.* administration

No.	TPE (h)^a^	ED_50_ MES^b^ (mg/kg)	ED_50_ *sc*PTZ^b^ (mg/kg)	TD_50_ NT^b^ (mg/kg)	PI (TD_50_/ED_50_)^c^
**20**	2	52.30 (46.38–60.22)	ND	>500	>9.56 (MES)
Valproic acid^d^	1	485 (324–677)	646 (466–869)	784 (503–1176)	1.6 (MES) 1.2 (*sc*PTZ)
Phenytoin^d^	1	28.10 (20.7–35.2)	>500	>100	>3.6 (MES)

*ND* not determined

^a^Time to peak effect

^b^Results are presented as mean ± SEM at 95 % confidence limit (*MES* maximal electroshock test, *scPTZ* subcutaneous pentylenetetrazole test, *NT* neurotoxicity, rotarod test)

^c^Protective index (TD_50_/ED_50_)

^d^Reference antiepileptic drugs tested by use of ADD Program procedures in NIH/NINDS

The quantitative data showed that **20** had higher ED_50_ than phenytoin (MES test), however, was distinctly more potent than valproic acid in this seizure model. It should be emphasized that in both cases **20** revealed more favorable protective indexes in comparison with mentioned model AEDs. Furthermore, substance **20** was almost twice weaker in the MES test than model succinimides presented in Fig. 2. These results support strongly crucial role of succinimide ring for anticonvulsant activity.

In the next step of pharmacological investigations, four compounds **12**, **19**, **20** and **24** were chosen for the evaluation of anticonvulsant activity in the psychomotor 6-Hz test (Table 4). The selection was made randomly as a part of the search of molecules providing anti-6-Hz protection among chemically diversified compounds pursued in the NIH/NINDS. It should be noticed that 6-Hz screen has been validated as a model of therapy-resistant epilepsy (Barton *et al*., 2001). Furthermore, the 6-Hz stimulation is suggested to be capable for identifying anti-seizure agents with a novel spectrum of activity and unknown mechanism of anticonvulsant action. One example supporting this hypothesis is provided by levetiracetam, which has demonstrated efficacy in refractory human partial epilepsies. It was found to be inactive against classical MES and PTZ seizures even at high doses, whereas showed high effectiveness in the 6-Hz psychomotor seizure model of partial epilepsy (Rogawski, 2006). Thus, it is suggested that the 6-Hz model might be capable for identifying anti-seizure agents with a novel spectrum of activity and unknown mechanism of anticonvulsant action.

**Table 4:** Anticonvulsant activity—*i.p.* psychomotor seizure test in mice (6-Hz)

No.	Intraperitoneal injection into mice^a^				
	0.25 h	0.5 h	1 h	2 h	4 h
**12**	3/4	3/4	0/4	0/4	0/4
**19**	0/4	0/4	0/4	0/4	1/4
**20**	1/4	0/4	0/4	1/4	1/4
**24**	4/4	2/4	3/4	0/4	0/4

Current 32 mA

^a^Dose of 100 mg/kg was administrated intraperitoneally. The data indicate: number of mice protected/number of mice tested

The results obtained showed the most potent activity for morpholine derivative **24** which protected 100 % of mice at 0.25 h, 75 % of animals at 1 h and 50 % of animals at 0.5 h at a dose of 100 mg/kg after (*i.p.*). Compound **12** protected 75 % of animals at 0.25 and 0.5 h. The other compounds **19** and **20** were less active and protected 25 % of animals at 4 h (**19**) as well as at 0.25, 2 and 4 h (**20**). None of these compounds caused motor impairment during mentioned studies.

### In vitro sodium channels radioligand binding studies

Intensive studies into the physiological and biochemical events taking place during epileptic seizures have provided insight into the molecular mechanisms by which these might be controlled. The fundamental role in establishing and regulating excitability of CNS neurons as well as suppression of seizures is ascribed to voltage-gated sodium channels (VGSCs) (Meldrum and Rogawski, 2007). Thus, the brain sodium channels are the molecular targets of numerous chemically diverse AEDs from which phenytoin and carbamazepine are the most representative (Rogawski and Löscher, 2004; Liu *et al*., 2003; Bialer *et al*., 2009). Using radioligand binding techniques, Catterall and associates have found an allosteric interaction between these drugs and the batrachotoxin (BTX) binding site of sodium channels from rat brain (Willow and Catterall, 1982; Catterall *et al*., 1981). Phenytoin and carbamazepine block the influx of sodium into rat brain synaptosomes elicited by BTX, which activates sodium channels (Willow *et al*., 1984). Electrophysiological studies of neuroblastoma cells demonstrate a frequency- and voltage-dependent blockade of sodium currents by both of these anticonvulsants (Willow *et al*., 1985; Matsuki *et al*., 1984). These findings suggest that blockade of sodium channel activity by these agents underlies their anticonvulsant actions. It should be stressed here that such mode of action is characteristic for compounds active in the MES seizure test in animals. Due to activity of reported compounds in the MES seizures, for the most active molecule **20,** the binding assays for Na+ channel (site 2) were performed using the [^3^H] batrachotoxin as radioligand (Brown, 1986). Compound binding was calculated as a percent inhibition of the binding of a radioactively labeled ligand. The inhibition values for **20** were obtained in four concentrations, 1, 10, 100, and 500 μM. All experiments were performed in duplicate. The results are given in Table 5.

**Table 5:** In vitro Na+ channel (site 2) binding assays

No.	Concentration (μM)	% Inhibition of control specific binding^a^
**20**	1	4.2
	10	7.5
	100	10.8
	500	46.7

^a^Compounds were each evaluated in synaptoneurosomal preparations from rat cerebral cortex as inhibitors of the specific binding of [^3^H]BTX to the voltage-sensitive sodium channel. Results showing an inhibition higher than 50 % are considered to represent significant effects of the test compounds; results showing an inhibition between 25 and 50 % are indicative of weak to moderate effects; results showing an inhibition lower than 25 % are not considered significant and mostly attributable to variability of the signal around the control level

As it can be seen, compound **20** was observed as a moderate binder to the neuronal voltage-sensitive sodium channel at the highest concentration—500 μM. This result may suggest different mechanism of anticonvulsant action than only influence on VGSCs.

## Experimental

### Chemistry

All the chemicals and solvents were purchased from Sigma-Aldrich (St. Louis, USA) and were used without further purification. Melting points (mp.) were determined in open capillaries on a Büchi 353 melting point apparatus (Büchi Labortechnik, Flawil, Switzerland) and are uncorrected. HPLC analyses were run on a HPLC Waters^®^ 2695 Separation Module equipped with photodiode array detector (Waters^®^ 2998). A Chromolith RP–18 SpeedROD column (4.6 × 50 mm) was used. Conditions applied were as follows: eluent A (water/0.1 % TFA), eluent B (acetonitrile/0.1 % TFA), flow rate 5 ml/min, gradient of 0–100 % B over 3 min were used, and injection volume was 10 µl. Standard solutions (1 mg/ml) of each compound were prepared in analytical grade acetonitrile and analyzed at wavelengths 214 and 254 nm. Retention times (*R*_t_) are given in minutes. Elemental analysis for C, H, and N was carried out by a micro method using the elemental Vario EI III Elemental analyzer (Hanau, Germany). The results of elemental analyses were within ±0.4 % of the theoretical values. ^1^H NMR,^13^C NMR and ^19^F NMR spectra were obtained in a Varian Mercury spectrometer (Varian Inc., Palo Alto, CA, USA), in CDCl_3_ operating at 300 MHz (^1^H NMR), 75 MHz (^13^C NMR) and 282 MHz (^19^F NMR). Chemical shifts are reported in *δ* values (ppm) relative to TMS *δ* = 0 (^1^H), as internal standard. The *J* values are expressed in Hertz (Hz). Signal multiplicities are represented by the following abbreviations: s (singlet), brs (broad singlet), d (doublet), ddd (double double doublet), t (triplet) and m (multiplet). The mass spectra were obtained on Waters ACQUITY™ TQD system with the TQ detector (Waters, Milford, USA). The ACQUITY UPLC BEH C18, 1.7 µm, 2.1 × 50 mm column was used (Waters, Milford, USA).

#### General procedure for preparation of intermediates **1** and **2**

A total of 0.05 mol of the 3-chloroaniline or 3-trifluoromethylaniline was dissolved in 30 ml of DCM and mixed with 5 ml 2 % aqueous solution of sodium hydroxide. Then, 0.05 mol of 2-chloroacetyl chloride dissolved in DCM (10 ml) was added dropwise over an hour. During that time, the reaction mixture was cooled with ice. After the acylating agent was added, stirring was continued for 2 h. Subsequently, the reaction mixture was extracted with saturated potassium bisulfate solution, water and brine. The organic layer was dried over the anhydrous sodium sulfate an distilled of on a rotary evaporator to obtain white crystalline powders.

##### *2-Chloro-N-(3-chlorophenyl)acetamide* (**1**)

White powdery crystals; mp 84–85 °C; HPLC: *R*_t_ = 1.089 min; ^1^H NMR (CDCl_3_): *δ* 4.21 (s, 2H, –CH_2_–), 7.41–7.50 (m, 2H, ArH), 7.58–7.78 (d, 1H, ArH, *J* = 7.70 Hz), 7.85 (s, 1H, ArH), 8.31 (brs, 1H, NH); ^13^C NMR (75 MHz, CDCl_3_): *δ* 43.3 (–CH_2_–), 115.6, 119.2, 124.2, 126.7, 131.6, 139.2 (6C_aromatic_), 168.8 (–CO–). ESI–MS: 204.1 (C_8_H_7_Cl_2_NO [M+H]^+^). Anal. Calcd for C_8_H_7_Cl_2_NO (204.05): C, 47.09; H, 3.46; N, 6.85. Found: C, 47.19; H, 3.55, N, 6.70.

##### *2-Chloro-N-[3-(trifluoromethyl)phenyl]acetamide* (**2**)

White powdery crystals; mp 72–73 °C; HPLC: *R*_t_ = 1.201 min; ^1^H NMR (300 MHz, CDCl_3_): *δ* 4.21 (s, 2H, –CH_2_–), 7.42–7.52 (m, 2H, ArH), 7.57–7.78 (m, 1H, ArH, *J* = 7.70 Hz), 7.85 (s, 1H, ArH), 8.33 (brs, 1H, NH); ^13^C NMR (75 MHz, CDCl_3_): *δ* 45.6 (–CH_2_–), 107.2 (–CF_3_), 117.1, 118.6, 120.5, 123.3, 126.4, 130.8, 135.6 (6C_aromatic_), 168.8 (–CO–). ESI–MS: 238.10 (C_9_H_7_ClF_3_NO [M+H]^+^). Anal. Calcd for C_9_H_7_ClF_3_NO (237.61): C, 45.49; H, 2.98; N, 5.89. Found: C, 45.55; H, 3.15, N, 5.88.

#### General procedure for the synthesis of final compounds **3–24**

A total of 0.002 mol of intermediate **1** or **2** and equimolar amount of corresponding secondary amine were dissolved in 10 ml of dry acetone. 0.006 mol of dry potassium carbonate and catalytic amount of potassium iodide were added, and the reaction mixture was stirred for about 24 h at a temperature of 60 °C (HPLC control). After this time, inorganic ingredients were filtered off and acetone was distilled off on a rotary evaporator. The crude products were obtained as colored oils. Compounds **3–22** and **24** were purified by column chromatography on Silica gel 60 (Merck, Darmstadt, Germany) using a dichloromethane–methanol (9: 0.7 *v/v*) mixture as a solvent system. Due to oily form, compounds **3**–**13**, **15**, **18**–**21**, **23** and **24** were converted into hydrochloride salts in anhydrous ethanol saturated with HCl gas. They were crystallized from anhydrous ethanol.

##### *N-(3-chlorophenyl)-2-(4-phenylpiperazin-1-yl)acetamide monohydrochloride* (**3**)

White powdery crystals; mp. 199–201 °C; HPLC: *R*_t_ = 1.357 min; ^1^H NMR (300 MHz, DMSO–*d*_6_): *δ* 3.06–3.93 (m, 8H, piperazine), 4.33 (s, 2H, –CH_2_–), 6.81–6.89 (m, 1H, ArH), 6.96–7.05 (m, 1H, ArH), 7.12–7.29 (m, 4H, ArH), 7.32–7.41 (m, 1H, ArH), 7.53–7.60 (m, 1H, ArH), 7.76–7.86 (m, 1H, ArH), 10.86 (brs, 1H, NH), 11.55 (brs, 1H, +NH); ^13^C NMR (75 MHz, DMSO–*d*_6_): *δ* 51.3 (–CH_2_–), 51.9 (–CH_2_–), 52.5 (–CH_2_–), 53.3 (–CH_2_–), 54.1 (–CH_2_–), 111.7, 117.0, 118.5, 123.6, 130.2, 130.9, 135.4, 140.6, 146.3 (12C_aromatic_), 172.1 (–CO–). ESI–MS: 330.4 (C_18_H_20_ClN_3_O [M+H]^+^). Anal. Calcd for C_18_H_21_Cl_2_N_3_O (366.28): C, 59.02; H, 5.78; N, 11.47. Found: C, 59.12; H, 5.90, N, 11.50.

##### *N-(3-chlorophenyl)-2-[4-(2-chlorophenyl)piperazin-1-yl]acetamide monohydrochloride* (**4**)

White powdery crystals; mp. 238–240 °C; HPLC: *R*_t_ = 1.456 min; ^1^H NMR (300 MHz, DMSO–*d*_6_): *δ* 3.09–3.50 (m, 8H, piperazine), 4.33 (s, 2H, –CH_2_–), 7.06–7.22 (m, 3H, ArH), 7.29–7.45 (m, 3H, ArH), 7.56 (ddd, 1H, ArH, *J* = 8.23, 1.92, 0.91 Hz), 7.82–7.88 (m, 1H, ArH), 10.87 (s, 1H, NH), 11.43 (brs, 1H, +NH); ^13^C NMR (75 MHz, DMSO–*d*_6_): *δ* 51.8 (–CH_2_–), 52.3 (–CH_2_–), 53.1 (–CH_2_–), 54.3 (–CH_2_–), 54.9 (–CH_2_–), 110.6, 116.8, 117.5, 118.3, 118.8, 124.5, 126.5, 130.3, 131.1, 136.8, 140.1, 144.2 (12C_aromatic_), 173.2 (–CO–). ESI–MS: 365.2 (C_18_H_19_Cl_2_N_3_O [M+H]^+^). Anal. Calcd for C_18_H_20_N_3_OCl_3_ (400.73): C, 53.95; H, 5.03; N, 10.49. Found: C, 54.03; H, 5.12; N, 10.55.

##### *N-(3-chlorophenyl)-2-[4-(4-chlorophenyl)piperazin-1-yl]acetamide monohydrochloride* (**5**)

White powdery crystals; mp. 217–219 °C; HPLC: *R*_t_ = 0.747 min; ^1^H NMR (300 MHz, DMSO–*d*_6_): *δ* 3.06–4.12 (m, 8H, piperazine), 4.30 (s, 2H, –CH_2_–), 6.96–7.04 (m, 2H, ArH), 7.15–7.20 (m, 1H, ArH), 7.24–7.31 (m, 2H, ArH), 7.37 (t, 1H, ArH, *J* = 8.11 Hz), 7.54 (d, 1H, ArH, *J* = 8.46 Hz), 7.83–7.84 (m, 1H, ArH), 10.71 (s, 1H, NH), 11.37 (brs, 1H, +NH); ^13^C NMR (75 MHz, DMSO–*d*_6_): *δ* 52.0 (–CH_2_–), 52.8 (–CH_2_–), 53.2 (–CH_2_–), 54.1 (–CH_2_–), 54.8 (–CH_2_–), 112.6, 114.5, 118.5, 119.3, 124.8, 126.6, 129.5, 135.7, 141.5, 146.4 (12C_aromatic_), 174.1 (–CO–). ESI–MS: 365.2 (C_18_H_19_Cl_2_N_3_O [M+H]^+^). Anal. Calcd for C_18_H_20_Cl_3_N_3_O (400.73): C, 53.95; H, 5.03; N, 10.49. Found: C, 53.98; H, 5.09; N, 10.53.

##### *N-(3-chlorophenyl)-2-[4-(2,3-dichlorophenyl)piperazin-1-yl]acetamide monohydrochloride* (**6**)

White powdery crystals; mp. 241–242 °C; HPLC: *R*_t_ = 1.601 min; ^1^H NMR (300 MHz, DMSO–*d*_6_): *δ* 3.14–3.78 (m, 8H, piperazine), 4.31 (s, 2H, –CH_2_–), 7.14–7.25 (m, 2H, ArH), 7.30–7.42 (m, 3H, ArH), 7.51–7.57 (m, 1H, ArH), 7.82–7.86 (m, 1H, ArH), 10.79 (brs, 1H, NH), 11.32 (brs, 1H, +NH); ^13^C NMR (75 MHz, DMSO–*d*_6_): *δ* 52.2 (–CH_2_–), 53.3 (–CH_2_–), 53.9 (–CH_2_–), 54.5 (–CH_2_–), 55.6 (–CH_2_–), 116.6, 117.1, 117.5, 118.7, 119.8, 124.7, 126.6, 129.3, 130.1, 136.5, 140.8, 147.1 (12C_aromatic_), 174.6 (–CO–). ESI–MS: 399.3 (C_18_H_18_Cl_3_N_3_O [M+H]^+^). Anal. Calcd for C_18_H_19_Cl_4_N_3_O (435.17): C, 49.68; H, 4.40; N, 9.66. Found: C, 49.75; H, 4.48; N, 9.74.

##### *N-(3-chlorophenyl)-2-[4-(2-fluorophenyl)piperazin-1-yl]acetamide monohydrochloride* (**7**)

White powdery crystals; mp. 224–225 °C; HPLC: *R*_t_ = 1.393 min; ^1^H NMR (300 MHz, DMSO–*d*_6_): *δ* 3.02–3.79 (m, 8H, piperazine), 4.32 (s, 2H, –CH_2_–), 6.98–7.21 (m, 5H, ArH), 7.33–7.42 (m, 1H, ArH), 7.56 (ddd, 1H, ArH, *J* = 8.23, 2.03, 1.03 Hz), 7.85 (t, 1H, ArH, *J* = 1.97 Hz), 10.84 (brs, 1H, NH), 11.45 (brs, 1H, +NH); ^13^C NMR (75 MHz, CDCl_3_): *δ* 50.8 (–CH_2_–), 51.9 (–CH_2_–), 53.2 (–CH_2_–), 54.4 (–CH_2_–), 55.8 (–CH_2_–), 114.2, 115.3, 116.5, 117.0, 117.8, 122.9, 125.4, 129.1, 129.6, 134.4, 138.5, 143.2 (12C_aromatic_), 171.9 (–CO–); ^19^F NMR (282 MHz, DMSO–*d*_6_): *δ* –122.92–(–122.83) (m, 1F). ESI–MS: 348.8 (C_18_H_19_ClFN_3_O [M+H]^+^). Anal. Calcd for C_18_H_20_Cl_2_FN_3_O (384.28): C, 56.26; H, 5.25; N, 10.93. Found: C, 56.30; H, 5.32; N, 11.04.

##### *N-(3-chlorophenyl)-2-[4-(4-fluorophenyl)piperazin-1-yl]acetamide monohydrochloride* (**8**)

White powdery crystals; mp. 214–216 °C; HPLC: *R*_t_ = 1.392 min; ^1^H NMR (300 MHz, DMSO–*d*_6_): *δ* 2.95–3.89 (m, 8H, piperazine), 4.32 (s, 2H, –CH_2_–), 6.96–7.19 (m, 4H, ArH), 7.36 (t, 2H, ArH, *J* = 8.11 Hz), 7.54–7.60 (m, 1H, ArH), 7.84–7.87 (m, 1H, ArH), 10.86 (brs, 1H, NH), 11.55 (brs, 1H, +NH); ^13^C NMR (75 MHz, DMSO–*d*_6_): *δ* 52.5 (–CH_2_–), 53.5 (–CH_2_–), 54.6 (–CH_2_–), 55.5 (–CH_2_–), 56.3 (–CH_2_–), 115.2, 116.4, 117.2, 117.9, 123.9, 125.8, 129.0, 134.6, 140.1, 145.1 (12C_aromatic_), 173.2 (–CO–); ^19^F NMR (282 MHz, DMSO–*d*_6_): *δ* –123.98 (s, 1F). ESI–MS: 348.7 (C_18_H_19_ClFN_3_O [M+H]^+^). Anal. Calcd for C_18_H_20_Cl_2_FN_3_O (384.28): C, 56.26; H, 5.25; N, 10.93. Found: C, 56.35; H, 5.34; N, 10.99.

##### *N-(3-chlorophenyl)-2-[4-[3-(trifluoromethyl)phenyl]piperazin-1-yl]acetamide monohydrochloride* (**9**)

White powdery crystals; mp. 199–200 °C; HPLC: *R*_t_ = 1.602 min; ^1^H NMR (300 MHz, DMSO–*d*_6_): *δ* 3.15–3.49 (m, 4H, piperazine), 3.57–3.70 (m, 2H, piperazine), 3.87–4.01 (m, 2H, piperazine), 4.33 (s, 2H, –CH_2_–), 7.10–7.18 (m, 2H, ArH), 7.21–7.30 (m, 2H, ArH), 7.32–7.49 (m, 2H, ArH), 7.54–7.60 (m, 1H, ArH), 7.85 (t, 1H, ArH, *J* = 2.00 Hz), 10.95 (brs, 1H, NH), 11.56 (brs, 1H, +NH); ^13^C NMR (75 MHz, DMSO–*d*_6_): *δ* 49.8 (–CH_2_–), 51.2 (–CH_2_–), 52.8 (–CH_2_–), 54.5 (–CH_2_–), 55.7 (–CH_2_–), 116.6, 118.0, 118.5, 119.1, 119.9, 121.2, 123.9, 125.6, 129.1, 131.2, 137.4, 139.9, 141.8 (–CF_3_, 12C_aromatic_), 170.5 (–CO–); ^19^F NMR (282 MHz, DMSO–*d*_6_): *δ* –61.07 (s, 3F). ESI–MS: 398.4 (C_19_H_19_ClF_3_N_3_O [M+H]^+^). Anal. Calcd for C_19_H_20_Cl_2_F_3_N_3_O (434.28): C, 52.55; H, 4.64; N, 9.68. Found: C, 52.68; H, 4.77; N, 9.80.

##### *N-(3-chlorophenyl)-2-(4-pyrimidin-2-ylpiperazin-1-yl)acetamide monohydrochloride* (**10**)

White powdery crystals; mp. 248–249 °C; HPLC: *R*_t_ = 1.138 min; ^1^H NMR (300 MHz, DMSO–*d*_6_): *δ* 3.11–3.74 (m, 8H, piperazine), 4.28 (s, 2H, –CH_2_–), 6.76 (t, 1H, ArH, *J* = 4.79 Hz), 7.16 (ddd, 1H, ArH, *J* = 8.02, 2.12, 1.01 Hz), 7.36 (t, 1H, ArH, *J* = 8.09 Hz), 7.52–7.58 (m, 1H, ArH), 7.84 (t, 1H, ArH, *J* = 1.97 Hz), 8.40–8.48 (m, 2H, ArH), 10.91 (brs, 1H, NH), 11.50 (brs, 1H, HCl); ^13^C NMR (75 MHz, DMSO–*d*_6_): *δ* 50.2 (–CH_2_–), 52.1 (–CH_2_–), 54.1 (–CH_2_–), 55.2 (–CH_2_–), 56.1 (–CH_2_–), 115.2, 117.9, 120.1, 123.3, 129.4, 133.1, 137.8, 158.5, 164.9 (10C_aromatic_), 170.8 (–CO–). ESI–MS: 332.7 (C_16_H_18_ClN_5_O [M+H]^+^). Anal. Calcd for C_16_H_19_Cl_2_N_5_O (368.26): C, 52.18; H, 5.20; N, 19.02. Found: C, 52.23; H, 5.29; N, 19.08.

##### *2-(4-Benzylpiperazin-1-yl)-N-(3-chlorophenyl)acetamide dihydrochloride* (**11**)

White powdery crystals; mp. 245–247 °C; HPLC: *R*_t_ = 1.254 min; ^1^H NMR (300 MHz, DMSO–*d*_6_): *δ* 3.21–3.78 (m, 8H, piperazine), 4.15 (s, 2H, –CH_2_–), 4.40 (s, 2H, –CH_2_–), 7.15 (ddd, 1H, ArH, *J* = 7.95, 2.05, 1.03 Hz), 7.35 (t, 1H, ArH, *J* = 8.08 Hz), 7.41–7.48 (m, 3H, ArH), 7.48–7.55 (m, 1H, ArH), 7.60–7.70 (m, 2H, ArH), 7.82 (t, 1H, ArH, *J* = 2.05 Hz), 9.94 (s, 1H, NH), 11.17 (brs, 1H, +NH), 12.11 (brs, 1H, +NH); ^13^C NMR (75 MHz, DMSO–*d*_6_): *δ* 50.3 (–CH_2_–), 51.5 (–CH_2_–), 52.7 (–CH_2_–), 53.8 (–CH_2_–), 54.4 (–CH_2_–), 65.7 (–CH_2_–, benzyl), 115.2, 116.9, 118.2, 124.8, 129.2, 130.4, 134.8, 136.5, 139.4 (12C_aromatic_), 174.6 (–CO–). ESI–MS: 344.8 (C_19_H_22_ClN_3_O [M+H]^+^). Anal. Calcd for C_19_H_24_Cl_3_N_3_O (416.77): C, 54.75; H, 5.80; N, 10.08. Found: C, 54.90; H, 5.94; N, 10.11.

##### *N-(3-chlorophenyl)-2-(4-methylpiperazin-1-yl)acetamide dihydrochloride* (**12**)

White powdery crystals; mp. 237–238 °C; HPLC: *R*_t_ = 0.958 min; ^1^H NMR (300 MHz, DMSO–*d*_6_) *δ* 2.80 (s, 3H, CH_3_), 3.25–3.79 (m, 8H, piperazine), 4.17 (s, 2H, –CH_2_–), 7.15 (ddd, 1H, ArH, *J* = 8.01, 2.12, 0.90 Hz), 7.35 (t, 1H, ArH, *J* = 8.09 Hz), 7.53 (ddd, 1H, ArH, *J* = 8.22, 2.04, 1.03 Hz), 7.83 (t, 1H, ArH, *J* = 1.92 Hz), 10.91 (brs, 1H, NH), 11.22 (brs, 1H, +NH), 11.89 (brs, 1H, +NH); ^13^C NMR (75 MHz, DMSO–*d*_6_): *δ* 32.8 (–CH_3_), 49.6 (–CH_2_–), 50.6 (–CH_2_–), 51.7 (–CH_2_–), 52.6 (–CH_2_–), 53.4 (–CH_2_–), 118.0, 120.5, 123.2, 125.6, 129.8, 137.9 (6C_aromatic_), 169.8 (–CO–). ESI–MS: 268.7 (C_13_H_18_ClN_3_O [M+H]^+^). Anal. Calcd for C_13_H_20_Cl_3_N_3_O (340.68): C, 45.83; H, 5.92; N, 12.33. Found: C, 45.88; H, 5.99; N, 12.41.

##### *N-(3-chlorophenyl)-2-morpholino-acetamide monohydrochloride* (**13**)

White powdery crystals; mp. 189–190 °C; HPLC: *R*_t_ = 1.067 min; ^1^H NMR (300 MHz, DMSO–*d*_6_): *δ* 1.59–1.88 (m, 4H, morpholine), 2.96–3.56 (m, 4H, morpholine), 4.15 (s, 2H, –CH_2_–), 7.13–7.19 (m, 1H, ArH), 7.37 (t, 1H, ArH, *J* = 8.08 Hz), 7.51–7.57 (m, 1H, ArH), 7.82–7.86 (m, 1H, ArH), 10.05 (brs, 1H, NH), 11.41 (brs, 1H, +NH); ^13^C NMR (75 MHz, DMSO–*d*_6_): *δ* 51.8 (–CH_2_–), 52.6 (–CH_2_–), 53.2 (–CH_2_–), 67.8 (–CH_2_–), 68.6 (–CH_2_–), 117.8, 120.3, 122.9, 125.8, 128.4, 138.1 (6C_aromatic_), 171.5 (–CO–). ESI–MS: 255.7 (C_12_H_15_Cl N_2_O_2_ [M+H]^+^). Anal. Calcd for C_12_H_16_ Cl_2_N_2_O_2_ (291.17): C, 49.50; H, 5.54; N, 9.62. Found: C, 49.54; H, 5.61; N, 9.68.

##### *2-(4-Phenylpiperazin-1-yl)-N-[3-(trifluoromethyl)phenyl]acetamide* (**14**)

White powdery crystals; mp. 152–153 °C; HPLC: *R*_t_ = 1.204 min; ^1^H NMR (300 MHz, CDCl_3_): *δ* 2.81 (t, 4H, piperazine, *J* = 5.0 Hz), 3.23 (s, 2H, –CH_2_–), 3.29 (t, 4H, piperazine, *J* = 5.0 Hz), 6.87–6.97 (m, 3H, ArH), 7.25–7.32 (m, 2H, ArH), 7.37 (d, 1H, ArH, *J* = 7.44 Hz), 7.46 (t, 1H, ArH, *J* = 8.6 Hz), 7.82 (s, 2H, ArH), 9.27 (s, 1H, NH); ^13^C NMR (75 MHz, CDCl_3_): *δ* 52.5 (–CH_2_–), 53.2 (–CH_2_–), 53.8 (–CH_2_–), 54.7 (–CH_2_–), 55.3 (–CH_2_–), 109.7, 116.1, 117.5, 118.3, 123.5, 128.2, 129.7, 136.4, 139.9, 147.4 (–CF_3_, 12C_aromatic_), 175.3 (–CO–); ^19^F NMR (282 MHz, CDCl_3_): *δ* –62.74 (s, 3F). ESI–MS: 364.0 (C_19_H_20_F_3_N_3_O [M+H]^+^). Anal. Calcd for C_19_H_20_F_3_N_3_O (363.38): C, 62.80; H, 5.55; N, 11.56. Found: C, 62.86; H, 5.62; N, 11.63.

##### *2-[4-(2-Chlorophenyl)piperazin-1-yl]-N-[3-(trifluoromethyl)phenyl]acetamide monohydrochloride* (**15**) 

White powdery crystals; mp. 239–241 °C; HPLC: *R*_t_ = 1.298 min; ^1^H NMR (300 MHz, DMSO–*d*_6_): *δ* 3.14–3.65 (m, 8H, piperazine), 4.33 (s, 2H, –CH_2_–), 7.10 (t, 1H, ArH, *J* = 7.59 Hz), 7.25 (d, 1H, ArH, *J* = 7.95 Hz), 7.33 (t, 1H, ArH, *J* = 7,71 Hz), 7.45 (t, 2H, ArH, *J* = 8.46 Hz), 7.62 (t, 1H, ArH, *J* = 7.95 Hz), 7.85 (d, 1H, ArH, *J* = 8.46 Hz), 8.14 (s, 1H, ArH), 10.78 (brs, 1H, NH), 11.47 (brs, 1H, +NH); ^13^C NMR (75 MHz, DMSO–*d*_6_): *δ* 52.6 (–CH_2_–), 52.9 (–CH_2_–), 53.5 (–CH_2_–), 54.3 (–CH_2_–), 54.9 (–CH_2_–), 109.8, 116.4, 117.6, 118.5, 119.8, 120.4, 123.2, 126.0, 130.6, 131.2, 135.7, 139.8, 143.9 (–CF_3_, 12C_aromatic_), 175.1 (–CO–); ^19^F NMR (282 MHz, DMSO–*d*_6_): *δ* –61.43 (s, 3F). ESI–MS: 397.8 (C_19_H_19_ClF_3_N_3_O [M+H]^+^). Anal. Calcd for C_19_H_20_Cl_2_F_3_N_3_O (434.28): C, 52.55; H, 4.64; N, 9.68. Found: C, 52.61; H, 4.79; N, 9.79.

##### *2-[4-(4-Chlorophenyl)piperazin-1-yl]-N-[3-(trifluoromethyl)phenyl]acetamide* (**16**)

White powdery crystals; mp. 170–171 °C; HPLC: *R*_t_ = 1.365 min; ^1^H NMR (300 MHz, CDCl_3_): *δ* 2.79 (t, 4H, piperazine, *J* = 5.00 Hz), 3.23 (s, 2H, –CH_2_–), 3.25 (t, 4H, piperazine, *J* = 4.87 Hz) 6.83–6.89 (m, 2H, ArH), 7.20–7.26 (m, 2H, ArH), 7.37 (d, 1H, ArH, *J* = 7.44 Hz), 7.46 (t, 1H, ArH, *J* = 8.6 Hz), 7.81 (s, 2H, ArH), 9.23 (s, 1H, NH); ^13^C NMR (75 MHz, CDCl_3_): *δ* 51.5 (–CH_2_–), 52.5 (–CH_2_–), 53.5 (–CH_2_–), 54.0 (–CH_2_–), 55.3 (–CH_2_–), 111.3, 116.5, 117.9, 118.6, 119.8, 124.5, 126.8, 129.9, 136.8, 141.7, 144.4 (–CF_3_, 12C_aromatic_), 173.5 (–CO–); ^19^F NMR (282 MHz, CDCl_3_): *δ* –62.74 (s, 3F). ESI–MS: 398.3 (C_19_H_19_ClF_3_N_3_O [M+H]^+^). Anal. Calcd for C_19_H_19_ClF_3_N_3_O (397.82): C, 57.36; H, 4.81; N, 10.56. Found: C, 57.49; H, 4.89; N, 10.63.

##### *2-[4-(2,3-Dichlorophenyl)piperazin-1-yl]-N-[3-(trifluoromethyl)phenyl]acetamide* (**17**)

White powdery crystals; mp. 174–175 °C; HPLC: *R*_t_ = 1.434 min; ^1^H NMR (300 MHz, CDCl_3_): *δ* 2.84 (t, 4H, piperazine, *J* = 4.74 Hz), 3.15 (t, 4H, piperazine, *J* = 4.48 Hz), 3.23 (s, 2H, –CH_2_–), 6.98–7.02 (m, 1H, ArH), 7.14–7.21 (m, 2H, ArH), 7.37 (d, 1H, ArH, *J* = 7.69 Hz), 7.46 (t, 1H, ArH, *J* = 7.95 Hz), 7.79 (s, 1H, ArH), 7.87 (d, 1H, ArH, *J* = 8.2 Hz), 9.28 (brs, 1H, NH); ^13^C NMR (75 MHz, CDCl_3_): *δ* 53.2 (–CH_2_–), 54.0 (–CH_2_–), 54.8 (–CH_2_–), 55.5 (–CH_2_–), 56.7 (–CH_2_–), 115.8, 117.0, 117.8, 118.5, 119.5, 120.6, 124.3, 126.2, 129.5, 131.8, 137.5, 141.1, 146.2 (–CF_3_, 12C_aromatic_), 175.8 (–CO–); ^19^F NMR (282 MHz, CDCl_3_): *δ* –62.70 (s, 3F). ESI–MS: 432.1 (C_19_H_18_Cl_2_F_3_N_3_O [M+H]^+^). Anal. Calcd for C_19_H_18_Cl_2_F_3_N_3_O (432.27): C, 52.79; H, 4.20; N, 9.72. Found: C, 52.90; H, 4.28; N, 9.83.

##### *2-[4-(2-Fluorophenyl)piperazin-1-yl]-N-[3-(trifluoromethyl)phenyl]acetamide monohydrochloride* (**18**)

White powdery crystals; mp. 228–231 °C; HPLC: *R*_t_ = 1.247 min; ^1^H NMR (300 MHz, DMSO–*d*_6_): *δ* 3.21–3.63 (m, 8H, piperazine), 4.30 (s, 2H, –CH_2_–), 7.00–7.20 (m, 5H, ArH), 7.48 (d, 1H, ArH, *J* = 7.69 Hz), 7.61 (t, 1H, ArH, *J* = 8.07 Hz), 7.83 (d, 1H, ArH, *J* = 8.46 Hz), 10.61 (brs, 1H, NH), 11,35 (brs, 1H, +NH); ^13^C NMR (75 MHz, DMSO–*d*_6_): *δ* 51.7 (–CH_2_–), 52.9 (–CH_2_–), 53.5 (–CH_2_–), 54.4 (–CH_2_–), 55.5 (–CH_2_–), 111.0, 114.2, 115.9, 116.3, 117.0, 119.8, 121.7, 125.1, 128.8, 129.9, 133.8, 140.5, 147.1 (–CF_3_, 12C_aromatic_), 176.6 (–CO–); ^19^F NMR (282 MHz, DMSO–*d*_6_): *δ* –122.93–(–122.84) (m, 1F), –61.43 (s, 3F). ESI–MS: 381.9 (C_19_H_19_F_4_N_3_O [M+H]^+^). Anal. Calcd for C_19_H_20_ClF_4_N_3_O (417.82): C, 54.62; H, 4.82; N. 10.06. Found: C, 54.67; H, 4.92; N, 10.15.

##### *2-[4-(4-Fluorophenyl)piperazin-1-yl]-N-[3-(trifluoromethyl)phenyl]acetamide monohydrochloride* (**19**)

White powdery crystals; mp. 204–206 °C; HPLC: *R*_t_ = 1.219 min; ^1^H NMR (300 MHz, DMSO–*d*_6_): *δ* 3.13–3.69 (m, 8H, piperazine), 4.32 (s, 2H, –CH_2_–), 6.98–7.12 (m, 4H, ArH), 7.48 (d, 1H, ArH, *J* = 7.69 Hz), 7.60 (t, 1H, ArH, *J* = 7.95 Hz), 7.84 (d, 1H, ArH, *J* = 8.46 Hz), 8.14 (s, 1H, ArH), 10,72 (brs, 1H, NH), 11,48 (s, 1H, +NH); ^13^C NMR (75 MHz, DMSO–*d*_6_): *δ* 52.1 (–CH_2_–), 53.0 (–CH_2_–), 53.9 (–CH_2_–), 54.5 (–CH_2_–), 55.3 (–CH_2_–), 112.2, 115.8, 117.5, 118.6, 120.7, 124.4, 125.9, 129.2, 135.7, 141.9, 146.8 (–CF_3_, 12C_aromatic_), 175.3 (–CO–); ^19^F NMR (282 MHz, DMSO–*d*_6_): *δ* –124.07 (s, 1F), –61.42 (s, 3F). ESI–MS: 381.8 (C_19_H_19_F_4_N_3_O [M+H]^+^). Anal. Calcd for C_19_H_20_ClF_4_N_3_O (417.82): C, 54.62; H, 4.82; N, 10.06. Found: C, 54.67;, 4.93; N, 10.13.

##### *N-[3-(Trifluoromethyl)phenyl]-2-[4-[3-(trifluoromethyl)phenyl]piperazin-1-yl]acetamide monohydrochloride* (**20**)

White powdery crystals; mp. 189–190 °C; HPLC: *R*_t_ = 1.437 min; ^1^H NMR (300 MHz, DMSO–*d*_6_): *δ* 3.13–3.69 (m, 8H, piperazine), 4.33 (s, 2H, –CH_2_–), 7.10–7.18 (m, 2H, ArH), 7.21–7.30 (m, 2H, ArH), 7.32–7.49 (m, 2H, ArH), 7.54–7.60 (m, 1H, ArH), 7.85 (t, 1H, ArH, *J* = 2.00 Hz), 10.61 (brs, 1H, NH), 11.46 (brs, 1H, +NH); ^13^C NMR (75 MHz, DMSO–*d*_6_): 52.2 (–CH_2_–), 52.9 (–CH_2_–), 53.8 (–CH_2_–), 55.7 (–CH_2_–), 56.8 (–CH_2_–), 115.2, 117.8, 118.6, 119.2, 119.9, 120.5, 121.6, 124.0, 126.3, 129.8, 131.7, 139.4, 142.2, 148.8 (–CF_3_, –CF_3_, 12C_aromatic_), 177.2 (–CO–); ^19^F NMR (282 MHz, DMSO–*d*_6_): *δ* –61.41 (s, 3F), –61.35 (s, 3F). ESI–MS: 431.4 (C_20_H_19_F_6_N_3_O [M+H]^+^). Anal. Calcd for C_20_H_20_ClF_6_N_3_O (467.84): C, 51.35; H, 4.31; N, 8.98. Found: C, 51.49; H, 4.44; N, 9.04.

##### *2-(4-Pyrimidin-2-ylpiperazin-1-yl)-N-[3-(trifluoromethyl)phenyl]acetamide monohydrochloride* (**21**)

White powdery crystals; mp. 149–150 °C; HPLC: *R*_t_ = 1.147 min; ^1^H NMR (300 MHz, DMSO–*d*_6_): *δ* 3.14–3.80 (m, 8H, piperazine), 4.26 (s, 2H, –CH_2_–), 6.75 (t, 1H, ArH, *J* = 4.87 Hz), 7.48 (d, 1H, ArH, *J* = 7.18 Hz), 7.61 (t, 1H, ArH, *J* = 8.08 Hz), 7.78–7.82 (m, 1H, ArH), 8.12 (s, 1H, ArH), 8.44 (d, 2H, ArH, *J* = 4.88 Hz), 10.59 (s, 1H, NH), 11.26 (brs, 1H, +NH); ^13^C NMR (75 MHz, DMSO–*d*_6_): *δ* 51.4 (–CH_2_–), 52.5 (–CH_2_–), 53.3 (–CH_2_–), 54.1 (–CH_2_–), 55.2 (–CH_2_–), 113.1, 118.8, 120.1, 121.3, 124.5, 129.9, 132.6, 139.8, 157.9, 167.8 (–CF_3_, 10C_aromatic_), 174.2 (–CO–); ^19^F NMR (282 MHz, DMSO–*d*_6_): *δ* –61.42 (s, 3F). ESI–MS: 366.0 (C_17_H_18_F_3_N_5_O [M+H]^+^). Anal. Calcd for C_17_H_19_ClF_3_N_5_O (401.81): C, 50.82; H, 4.77; N, 17.43. Found: C, 50.97; H, 4.82; N, 17.55.

##### *2-(4-Benzylpiperazin-1-yl)-N-[3-(trifluoromethyl)phenyl]acetamide* (**22**)

White powdery crystals; mp. 115–116 °C; HPLC: *R*_t_ = 1.147 min; ^1^H NMR (300 MHz, CDCl_3_): *δ* 2.55–2.67 (m, 8H, piperazine), 3.15 (s, 2H, –CH_2_–), 3.56 (s, 2H, –CH_2_–), 7.24–7.37 (m, 6H, ArH), 7.45 (t, 1H, ArH, *J* = 7.95 Hz), 7.79–7.83 (m, 2H, ArH), 9.29 (s, 1H, NH); ^13^C NMR (75 MHz, CDCl_3_): *δ* 49.2 (–CH_2_–), 50.1 (–CH_2_–), 51.2 (–CH_2_–), 52.3 (–CH_2_–), 53.5 (–CH_2_–), 65.8 (–CH_2_–), 114.8, 117.5, 118.6, 120.2, 125.3, 128.9, 130.9, 133.7, 135.8, 138.3 (–CF_3_, 12C_aromatic_), 175.5 (–CO–); ^19^F NMR (282 MHz, CDCl_3_): *δ* –62.70 (s, 3F). ESI–MS: 378.4 (C_20_H_22_F_3_N_3_O [M+H]^+^). Anal. Calcd for C_20_H_22_F_3_N_3_O (377.40): C, 63.65; H, 5.88; N, 11.13. Found: C, 63.73; H, 5.96; N, 11.28.

##### *2-[4-(2-Hydroxyethyl)piperazin-1-yl]-N-[3-(trifluoromethyl)phenyl]acetamide dihydrochloride* (**23**)

White powdery crystals; mp. 210–212 °C; HPLC: *R*_t_ = 0.841 min; ^1^H NMR (300 MHz, DMSO–*d*_6_): *δ* 3.23–3.94 (m, 15H; 8H: piperazine, 4H: –C2H4–, 2H: –CH2–, 1H: –OH), 7.55 (d, 2H, ArH, *J* = 7.42 Hz), 7.58 (t, 1H, ArH, *J* = 7.95 Hz), 7.84 (d, 1H, ArH, *J* = 8,2 Hz), 9.75 (brs, 1H, NH), 10.97 (brs, 1H, +NH), 11.89 (brs, 1H, +NH); ^13^C NMR (75 MHz, DMSO–*d*_6_): *δ* 47.5 (–CH_2_–), 48.6 (–CH_2_–), 49.9 (–CH_2_–), 51.1 (–CH_2_–), 52.4 (–CH_2_–), 63.4 (–CH_2_–), 118.7, 119.6, 120.8, 123.1, 125.8, 131.6, 138.8 (–CF_3_, 6C_aromatic_), 170.9 (–CO–); ^19^F NMR (282 MHz, DMSO–*d*_6_): *δ* –61.41 (s, 3F). ESI–MS: 332.2 (C_15_H_20_F_3_N_3_O_2_ [M+H]^+^). Anal. Calcd for C_15_H_22_Cl_2_F_3_N_3_O_2_ (404.26): C, 44.57; H, 5.49; N, 10.39. Found: C, 44.65; H, 5.57; N, 10.48.

##### *2-Morpholino-N-[3-(trifluoromethyl)phenyl]acetamide monohydrochloride* (**24**)

White powdery crystals; mp. 185–186 °C; HPLC: *R*_t_ = 0.985 min; ^1^H NMR (300 MHz, DMSO–*d*_6_): *δ* 1.65–1.77 (m, 4H, morpholine), 3.14–3.48 (m, 4H, morpholine), 4.16 (s, 2H, –CH_2_–), 7.47 (d, 1H, ArH, *J* = 7.70 Hz), 7.59 (t, 1H, ArH, *J* = 7.94 Hz), 7.83 (d, 1H, ArH, *J* = 8.20 Hz), 8.12 (s, 1H, ArH), 9.96 (brs, 1H, NH), 11.42 (brs, 1H, +NH); ^13^C NMR (75 MHz, DMSO–*d*_6_): *δ* 50.3 (–CH_2_–), 51.5 (–CH_2_–), 52.2 (–CH_2_–), 67.5 (–CH_2_–), 67.6 (–CH_2_–), 117.6, 119.2, 121.0, 123.3, 125.8, 133.4, 137.9 (–CF_3_, 6C_aromatic_), 172.1 (–CO–); ^19^F NMR (282 MHz, DMSO–*d*_6_): *δ* –61.44 (s, 3F). ESI–MS: 289.2 (C_13_H_15_F_3_N_2_O_2_ [M+H]^+^). Anal. Calcd for C_13_H_16_ClF_3_N_2_O_2_ (324.72): C, 48.08; H, 4.97; N, 8.63. Found: C, 48.20; H, 5.02; N, 8.75.

### Pharmacology

#### In vivo studies

Compounds **3**–**24** were pharmacologically preevaluated within the Antiepileptic Drug Development (ADD) Program in Epilepsy Branch, Neurological Disorders Program, National Institute of the Neurological and Communicative Disorders and Stroke (NIH/NINDS), Rockville, MD, USA, by using procedures described elsewhere (Krall *et al*., 1978; Kupferberg, 1989).

Phase I of the in vivo studies involved three tests: MES, *sc*PTZ and rotarod test for NT. Male albino mice (*CF#1* strain, weighing 18–25 g) and male albino rats (*Sprague*–*Dawley)* were used as experimental animals. The animals were housed in metabolic cages and allowed free access to food and water. The compounds were injected intraperitoneally to mice as a suspension in 0.5 % methylcellulose/water mixture at a dose level 30, 100 or 300 mg/kg with anticonvulsant activity and neurotoxicity assessment at 0.5 and 4 h after administration. Promising derivatives from phase I underwent phase VIa in which they were administrated orally into rats at a fixed dose of 30 mg/kg for both MES and the rotarod toxicity determination at five pretreatment times: 0.25, 0.5, 1, 2, 4 h. Groups of eight mice or four rats are employed.

#### Maximal electroshock seizure test (MES)

In the MES screen, an electrical stimulus of 0.2 s in duration (50 mA in mice and 150 mA in rats) is delivered via corneal electrodes primed with an electrolyte solution containing an anesthetic agent. The endpoint is the tonic extension of the hind limbs. In the control groups, the procedures cause immediate hind limb tonic extension. Mice not displaying hind limb tonic extension are considered to be protected from seizures.

#### Subcutaneous pentylenetetrazole seizure test (scPTZ)

The *sc*PTZ test utilizes a dose of pentylenetetrazole (85 mg/kg in mice) that produces clonic seizures lasting for a period of at least 5 s in 97 % (CD_97_) of animals tested. PTZ is administered 0.5 and 4 h after injections of tested compounds, and observation is carried out for 30 min. In the control groups, the first episode of clonic convulsions is observed between 6 and 15 min of observation. The absence of clonic convulsions in the observed time period of 0.5 and 4 h is interpreted as the compound’s ability to protect against PTZ-induced seizures.

#### Neurotoxicity screening

The neurological toxicity test (NT) induced by a compound was detected in mice or rats using standardized rotarod test (Dunham and Miya, 1957). Untreated control mice or rats, when placed on the rod, can maintained their equilibrium for a prolonged time period. The acute motor impairment can be demonstrated by the inability of the animal to maintain equilibrium for 1 min in each of three successive trials.

#### The psychomotor 6-Hz model

The 6-Hz model is an alternative electroshock paradigm that uses low-frequency (6 Hz), long-duration (3 s) electrical stimulation. Corneal stimulation (0.2 ms duration monopolar rectangular pulses at 6-Hz for 3 s) was delivered by a constant-current device. During the stimulation, mice were manually restrained and released into the observation cage immediately after the current application. The seizures manifest in “stunned” posture associated with rearing, forelimb, automatic movements and clonus, twitching the vibrissae and Straub–tail. The duration of the seizure activity ranges from 60 to 120 s in untreated animals. At the end of the seizure, animals resume their normal exploratory behavior. The experimental end point is protection against the seizure. The animal is considered to be protected if it resumes its normal exploratory behavior within 10 s from the stimulation (Brown *et al*., 1953).

#### In vitro studies–sodium channel binding assay

The radioligand binding studies were performed commercially in Cerep Laboratories (Poitiers, France) using testing procedures described in detail elsewhere (Brown, 1986). The general information is listed in Table 6.

**Table 6:** Experimental conditions—Na+ channel (site 2) binding assays

Source	Ligand	Concentration	Kd	Nonspecific binding	Incubation	Detection method
Rat cerebral cortex	[^3^H] Batrachotoxin	10 nM	91 nM	Veratridine (300 μM)	60 min 37 °C	Scintillation counting

## Conclusion

In the current studies, the library of twenty-two new *N*-phenyl-2-(4-phenylpiperazin-1-yl)acetamide derivatives has been synthesized and evaluated for their anticonvulsant activity in the maximum electroshock (MES) and *sc*PTZ seizure tests. The results of in vivo pharmacological studies showed activity exclusively in the MES seizures especially for 3-(trifluoromethyl)anilide derivatives, whereas majority of 3-chloroanilide analogs were inactive. Several molecules showed activity in the 6-Hz screen that may indicate their potential usefulness in partial and therapy-resistant epilepsy. The pharmacophoric role of the pyrrolidine-2,5-dione ring for anticonvulsant properties was proved.
